# Supplementary material for: Rapid and reversible photoinduced switching of a rotaxane crystal
Source: Nat Commun. 2016 Nov 3;7:13321. doi: 10.1038/ncomms13321 (PMC5097158; doi:10.1038/ncomms13321)
Supplement: Supplementary Information — Supplementary Figures 1-12 and Supplementary Tables 1-2. [file ncomms13321-s1.pdf]

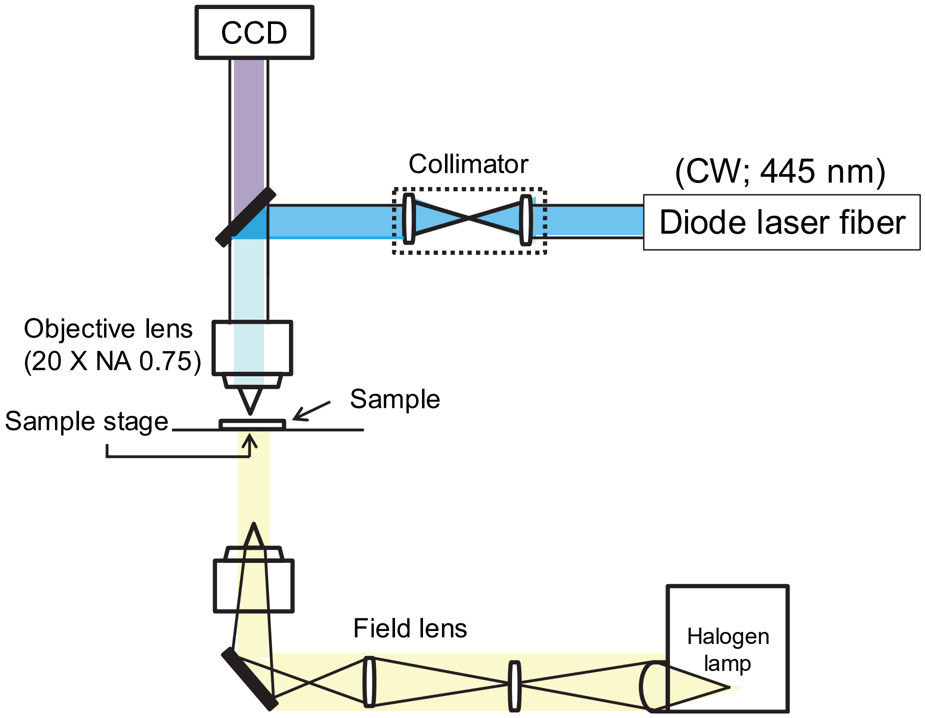


**Supplementary Figure 1 │A schematic diagram of the experimental setup**

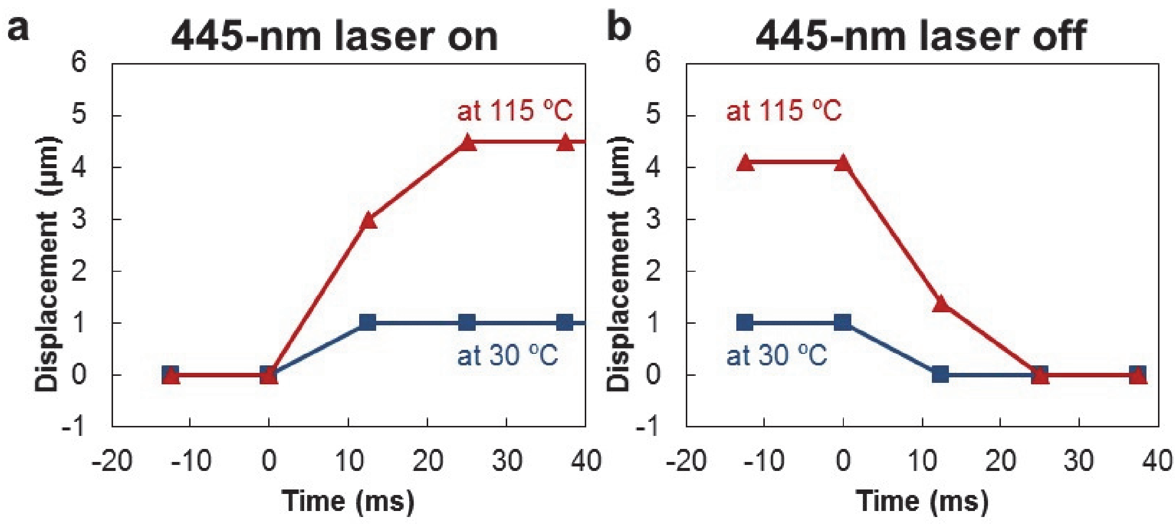


**Supplementary Figure 2 │Time dependence of the displacement of rotaxane crystal induced by laser. a,** Crystal expansion **(LT** to **LI)** was completed within 12 ms at 30 °C, and the reversible crystal-to-crystal phase transition (**LT** to **HT)** was completed within 25 ms at 115 °C. **b,** The crystal can return to its original size spontaneously on the same time scale. The measurements were performed using 445-nm focused laser irradiation of the crystal at 30 and 115 °C using laser powers of 6 and 3 mW, respectively.

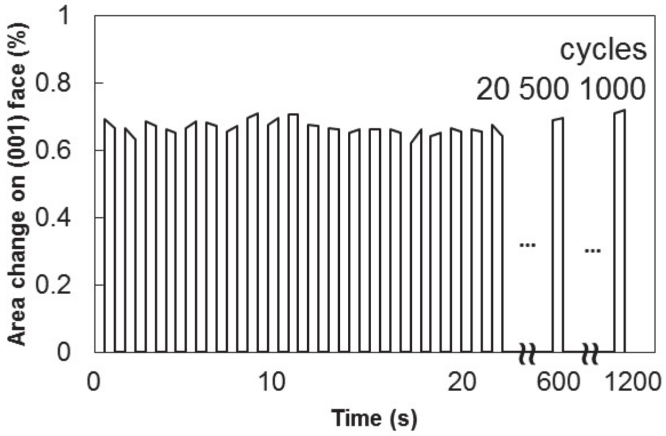


**Supplementary Figure 3 │Time dependence of the relative change in the top surface area [Miller index (001)] of the crystal.** Measurements were obtained after 11 mW 445 nm laser irradiation for 1.2 s intervals.

Thermographic analysis was conducted to analyze effect of the laser irradiation on the crystal temperature. The temperature increase was proportional to the laser power (Supplementary Figs. 4a, 4d). In the time-dependence measurements, the temperature change was completed within 60 ms from the start of the laser irradiation; no further increase in temperature was observed (Supplementary Figs. 4b, 4e). Turning the laser off causes the decrease of the temperature to the initial temperature within 80 ms (Supplementary Figs. 4c, 4f). Thus, laser irradiation (90 mW) at 115 ºC caused the temperature to rise to 130 ºC within 60 ms and then return to the initial temperature value within 80 ms after the turning the irradiation off (Fig. 2b and Supplementary Figs. 4c, 4f).

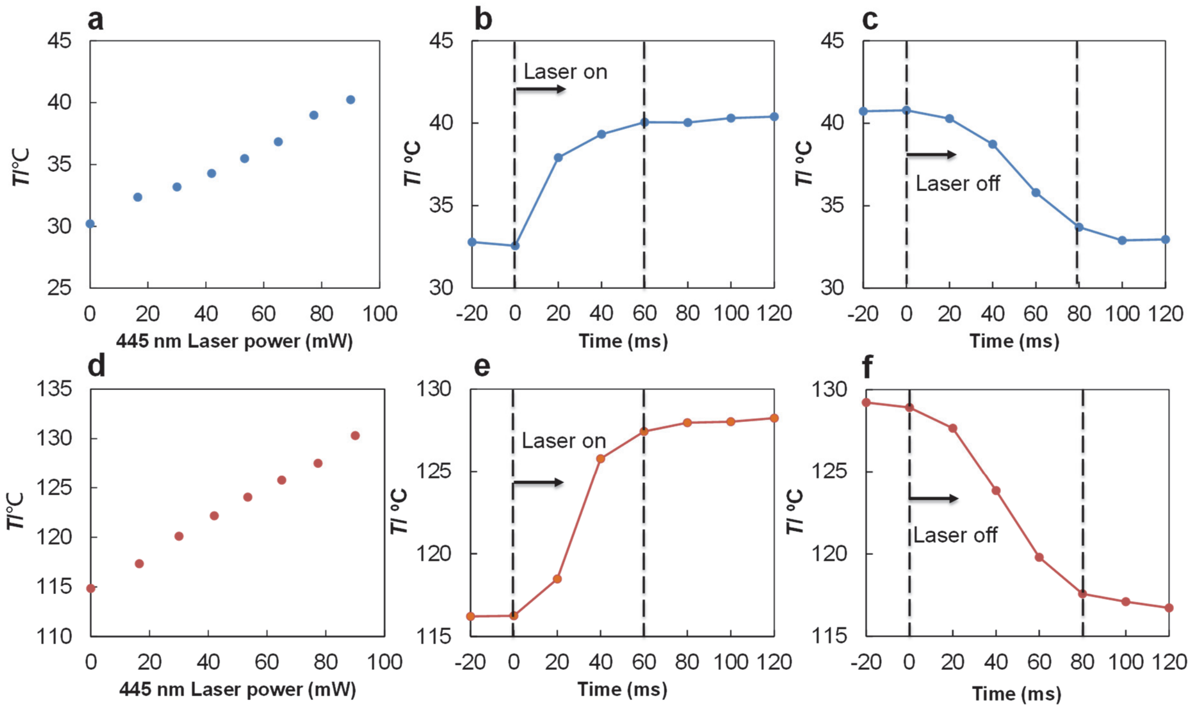


**Supplementary Figure 4│Time dependence of the temperature of the crystal induced by laser on and off. a, d,** Laser power dependence curve. **b, e,** Temperature change was completed within 60 ms of the start of laser irradiation at 445 nm. **c, f,** Temperature decreases to its initial value within 80 ms after the laser is switched off.

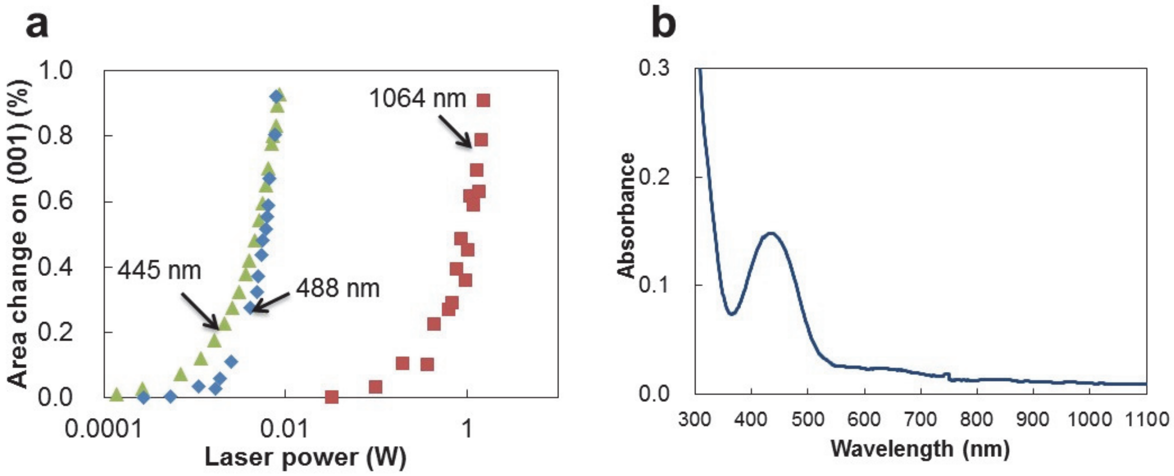


**Supplementary Figure 5 | Photo-response of the rotaxane crystal. a,** Laser power dependence of the change in the relative area of the (001) facet of the single crystal. **b,** UV–Vis absorption spectrum of the rotaxane in chloroform solution.

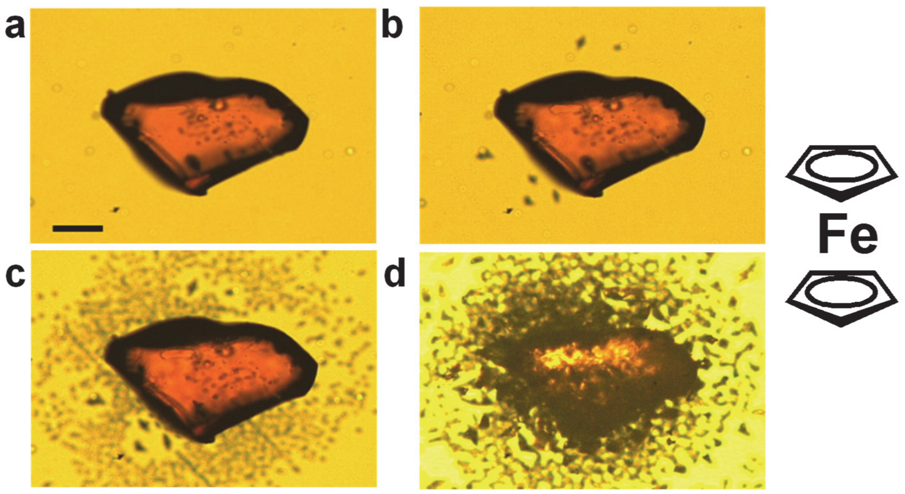


**Supplementary Figure 6 | Photo-response of ferrocene single crystal. a,** Before laser irradiation. **b,** Under 445-nm focused laser irradiation with the power of 1 mW. **c**, Under laser power of 2.5 mW. **d**, Under laser power of 4.5 mW. The scale bar, 50 µm.

Wide field irradiation of the crystals (crystal dimensions: 14.4 × 78.1 × 11.8 μm) was conducted by single-crystal X-ray crystallography (beam diameter was approximately 1 mm with beam power of 0–74 mW at 30 ºC).

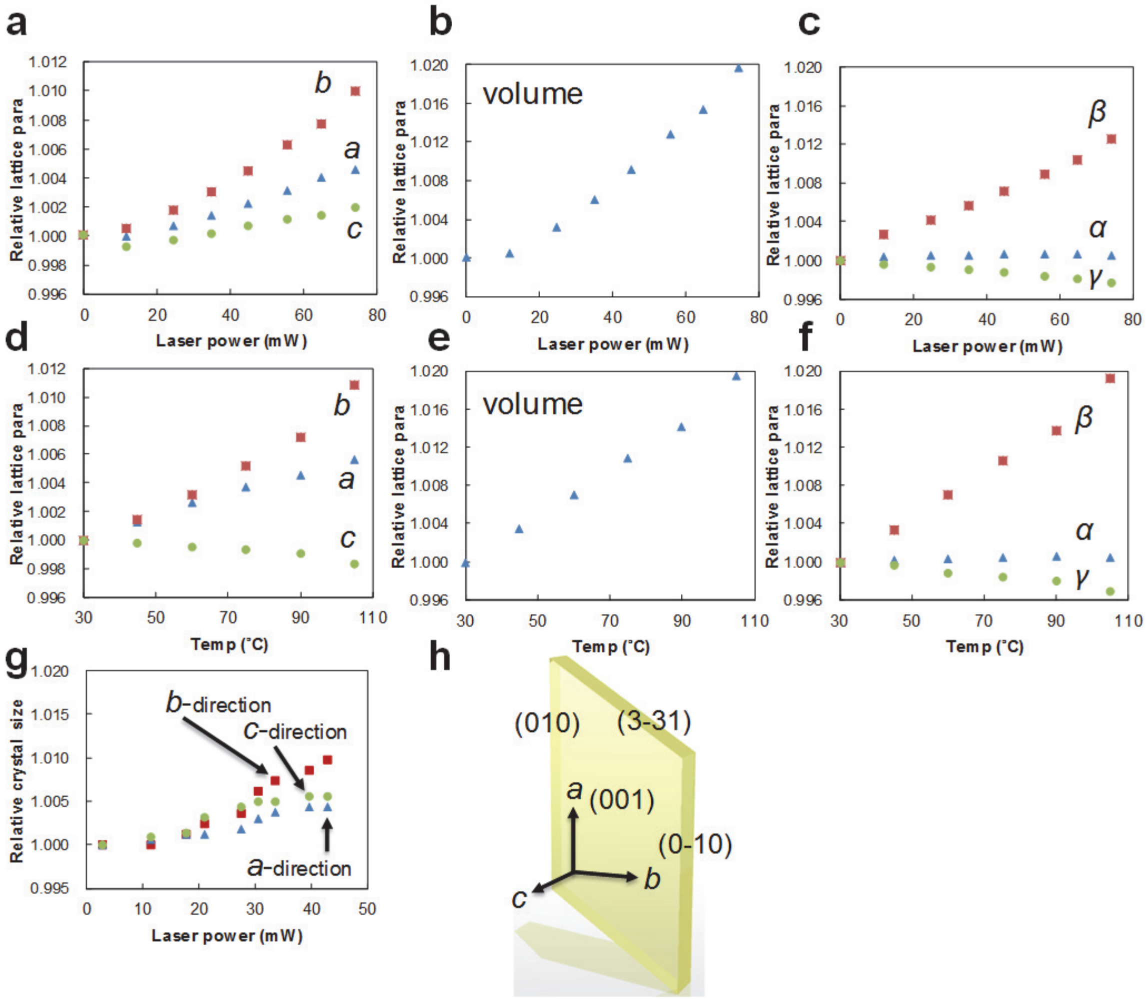


**Supplementary Figure 7 | Single-crystal X-ray crystallography. a,** Relative unit cell parameters *a*, *b*, *c* and **b,** Relative volume and **c,** Relative angles between the sides given by α, β, γ increased/decreased with power of 445-nm wide field laser irradiation. **d,** Relative unit cell parameters *a*, *b*, *c* and **e,** Relative volume and **f,** Relative angles between the sides given by α, β, γ increased/decreased with different temperature from 30 ºC to 105 ºC , respectively. **g.** The results observed for the change of crystal size by the laser irradiation at 30 ºC, which indicate that concerted mechanical motions of the rotaxane molecules lead to the expansion of the entire crystal. **h**, Schematic image of the rotaxane crystal showing unit axes and Miller indices.

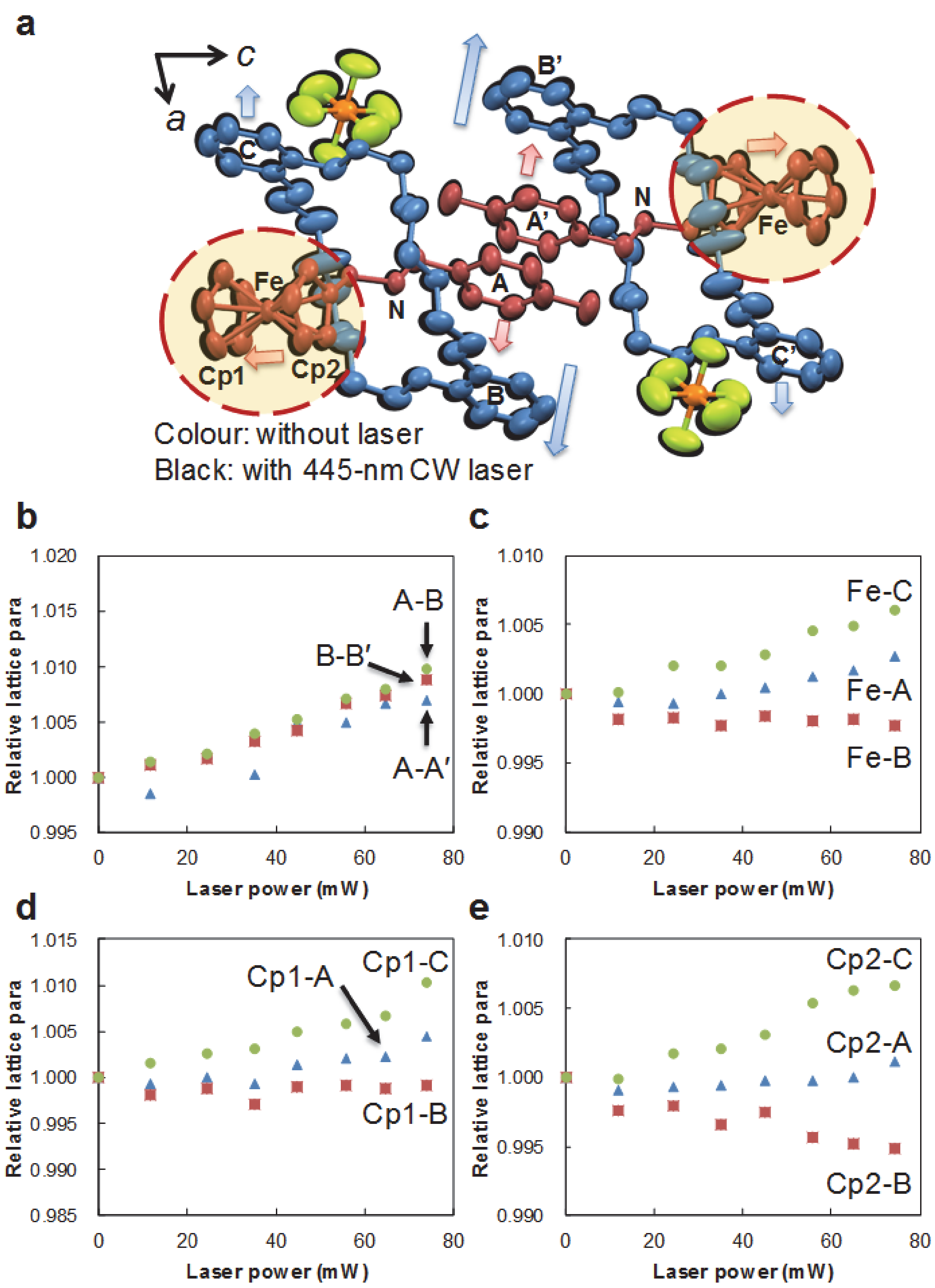


**Supplementary Figure 8 | Power dependence of the rotaxane relative lattice parameters. a,** Superimposed images of molecular structures of **LT** at 30 ºC (colour) and **LI** under 74 mW of 445-nm laser irradiation at 30 ºC (black) with 50% probability. **b**, **c**, **d**, **e**, Different relative inter/intra molecular distance changes along the three vectors (*a*, *b*, and *c*) that form the edges of the parallelepiped lattice of the single crystal as obtained by single-crystal X-ray crystallography.

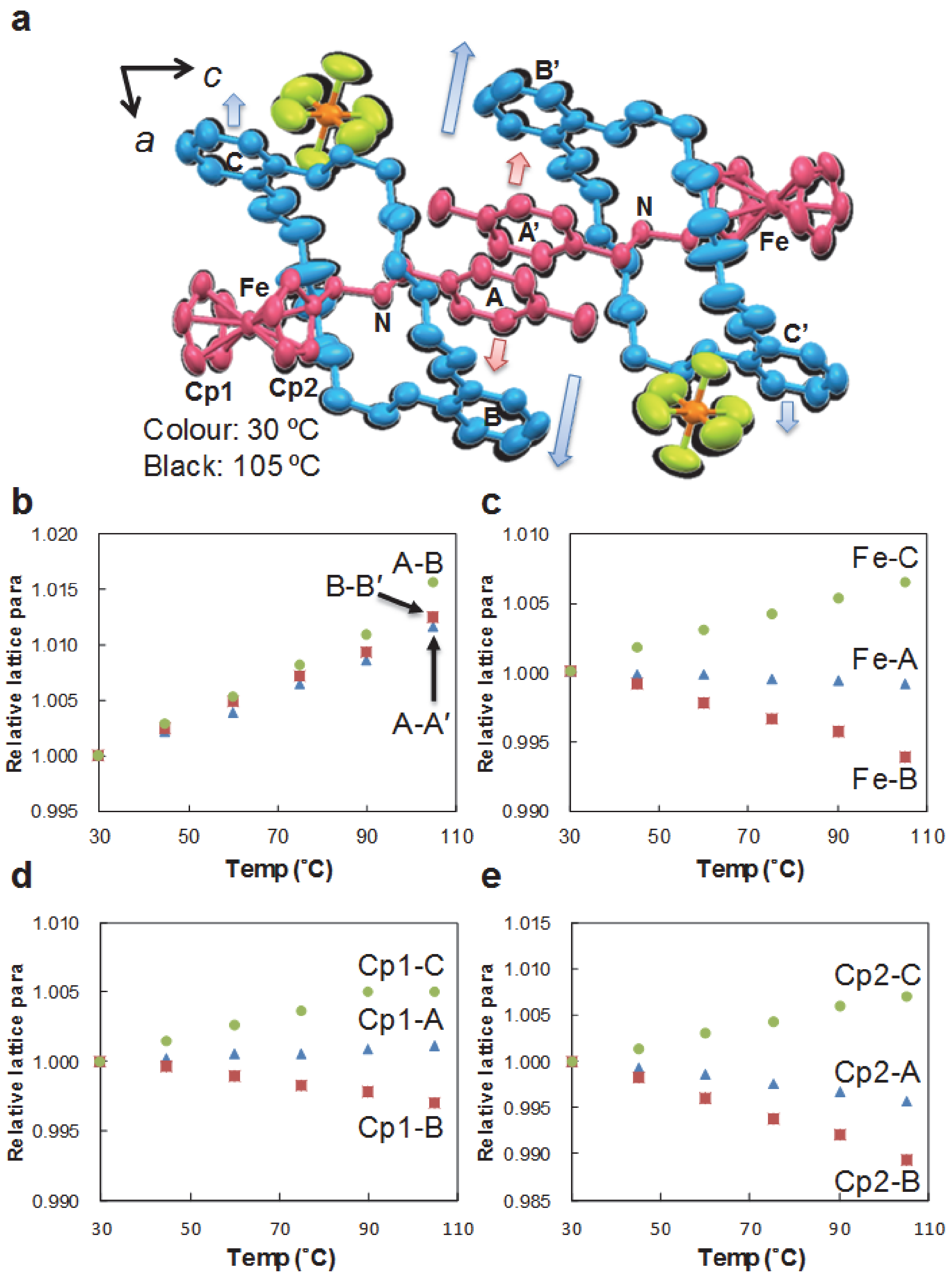


**Supplementary Figure 9│Temperature dependence of the rotaxane relative lattice parameters. a,** Superimposed images of molecular structures of **LT** at 30 ºC (colour) and the structure of same crystal at 105 ºC (black) with 50% probability. **b**, **c**, **d**, **e**, Different relative inter/intra molecular distance changes along the three vectors (*a*, *b*, and *c*) that form the edges of the parallelepiped lattice of the single crystal as obtained by single-crystal X-ray crystallography.

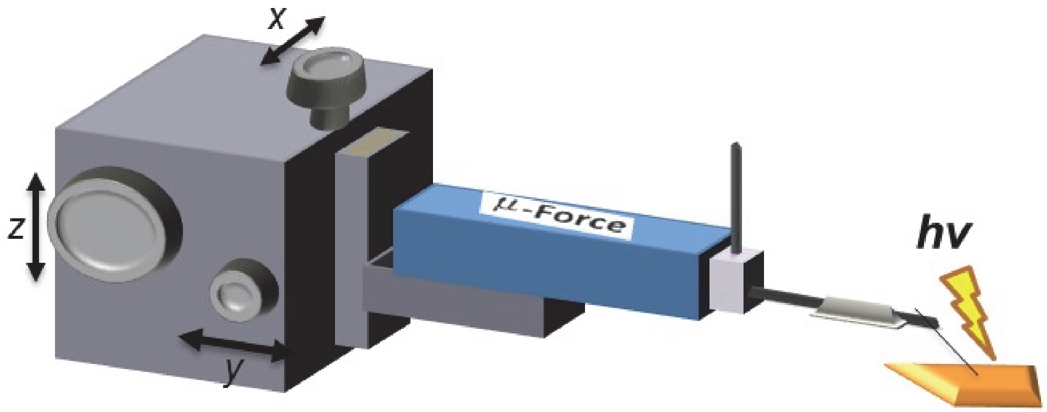


**Supplementary Figure 10│μ -Force detector.** This detector comprises two gauges on a cantilever beam with a microneedle attached at the tip as a detector.

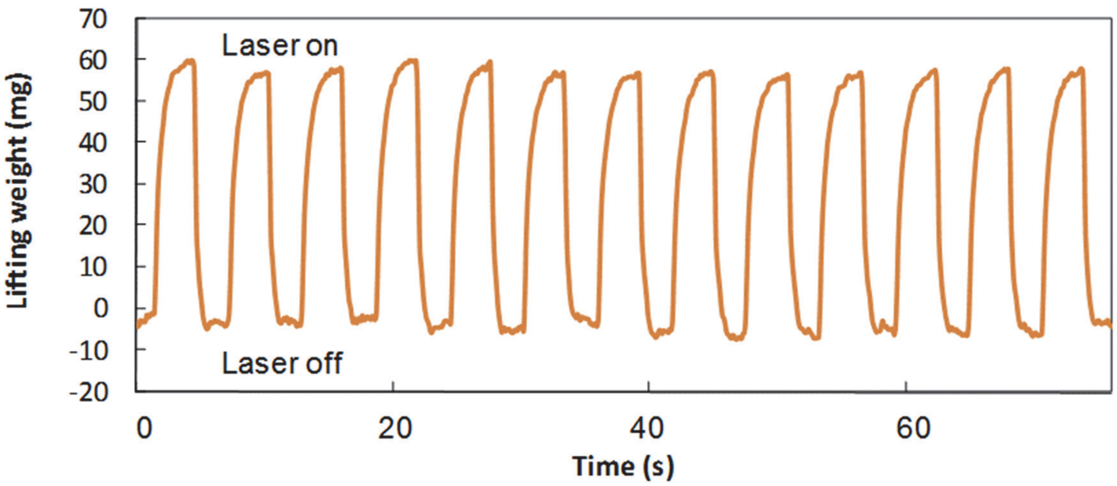


**Supplementary Figure 11│Time dependence of force detection.** Size expansion/contraction behavior of the rotaxane crystal induced by 445-nm laser irradiation was transduced into a mechanical force. The crystal can lift a weight of about 60 mg by turning the 15 mW laser irradiation on and off at 30 ºC.

The dependence of crystal birefringence ($\Delta n$) on the 445-nm laser power was studied using a crossed polarizer microscope and a Berek compensator.

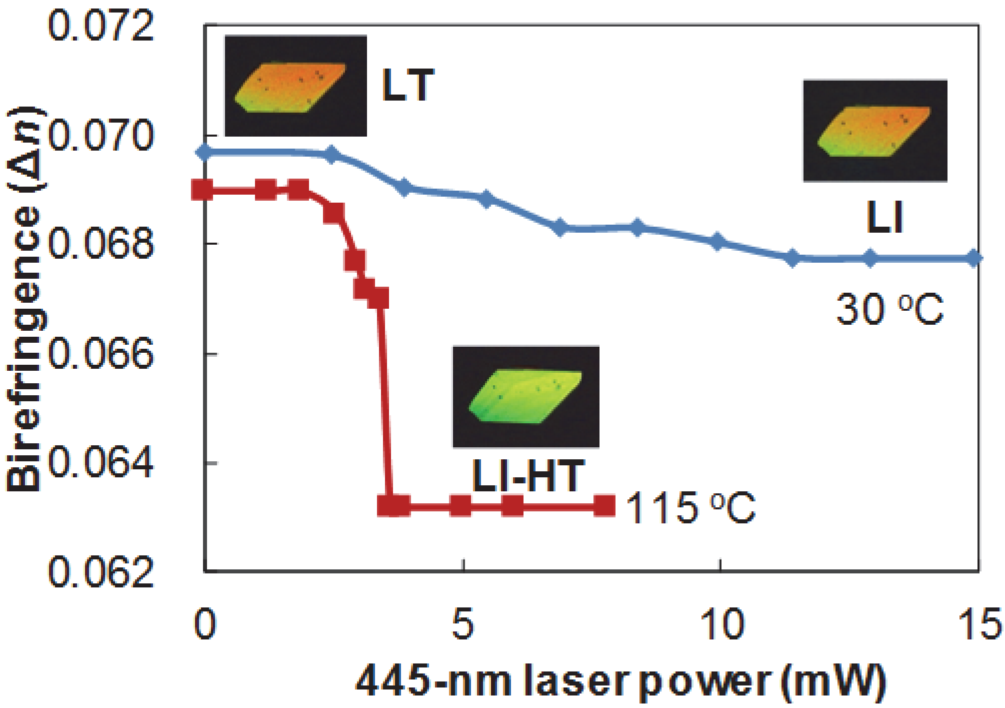


**Supplementary Figure 12 | Laser power dependence of the birefringence of the crystal at 30 °C and 115 °C.** At 30 °C, $\Delta n$ continuously changed from 0.070 to 0.068 as the laser power increased to 15 mW, whereas $\Delta n$ discontinuously decreased from 0.069 to 0.063 due to laser irradiation at 115 °C accompanied by a significant change of the interference colour. These changes correspond to the molecular structural change from **LT** to **LI** at 30 °C (Fig. 1, path I) and **LT** to **LI-HT** at 115 °C (Fig. 1, path II), respectively.

**Supplementary Table 1** │Crystal data and details of structure refinement of pseudorotaxanes with different power of laser irradiation.

| 445-nm laser power | **12 mW** | **25 mW** | **35 mW** | **45 mW** | **55 mW** | **65 mW** | **74 mW** |
|---|---|---|---|---|---|---|---|
| formula | $C_{43}H_{54}F_6FeNO_8P$ | | | | | | |
| molecular weight | 913.69 | | | | | | |
| crystal system | Triclinic | | | | | | |
| space group | P1bar (No.2) | | | | | | |
| *a*/ Å | 10.343(6) | 10.3504(7) | 10.3579(9) | 10.3662(12) | 10.3758(15) | 10.3815(18) | 10.391(2) |
| *b*/ Å | 11.1816(8) | 11.1958(9) | 11.210(12) | 11.2263(15) | 11.2462(19) | 11.262(2) | 11.288(3) |
| *c*/ Å | 19.4313(13) | 19.4394(14) | 19.4479(14) | 19.458(2) | 19.467(3) | 19.473(14) | 19.483(4) |
| $\alpha$/deg | 87.281(2) | 87.289(3) | 87.293(3) | 87.294(4) | 87.299(6) | 87.298(7) | 87.29(8) |
| $\beta$/deg | 79.707(2) | 79.822(3) | 80.940(4) | 80.055(5) | 80.198(6) | 80.314(7) | 80.485(9) |
| $\gamma$/deg | 88.818(2) | 88.798(2) | 88.772(3) | 88.745(4) | 88.715(5) | 88.684(6) | 88.65(8) |
| $V$/ Å$^3$ | 2208.4(3) | 2214.5(3) | 2220.7(4) | 2227.7(5) | 2235.7(6) | 2241.5(7) | 2250.8(9) |
| *Z* | 2 | 2 | 2 | 2 | 2 | 2 | 2 |
| $\mu$(MoKα) /cm$^{-1}$ | 4.54 | 4.53 | 4.52 | 4.50 | 4.49 | 4.48 | 4.46 |
| *F*(000) | 956 | 956 | 956 | 956 | 956 | 956 | 956 |
| *D*/g cm$^{-3}$ | 1.374 | 1.370 | 1.366 | 1.362 | 1.357 | 1.354 | 1.348 |
| crystal size/mm | 0.35x0.30x 0.10 | 0.35x0.30x 0.10 | 0.35x0.30x 0.10 | 0.35x0.30x 0.10 | 0.35x0.30x 0.10 | 0.35x0.30x 0.10 | 0.35x0.30x 0.10 |
| Unique reflections | 33186 | 33286 | 33313 | 33265 | 33261 | 33235 | 33407 |
| Used reflections [$I$>2.0σ($I$)] | 8858 | 8893 | 8901 | 8884 | 8891 | 8895 | 8934 |
| *R* | 0.0625 | 0.0658 | 0.0646 | 0.0661 | 0.0653 | 0.0648 | 0.0658 |
| *R*w | 0.1724 | 0.1863 | 0.1792 | 0.1848 | 0.1765 | 0.1800 | 0.1825 |
| GOF | 0.994 | 0.986 | 0.982 | 0.968 | 0.964 | 0.958 | 0.945 |

**Supplementary Table 2** │Crystal data and details of structure refinement of pseudorotaxanes at different temperature.

| Temp. | **30 °C** | **45 °C** | **60 °C** | **75 °C** | **90 °C** | **105 °C** |
|---|---|---|---|---|---|---|
| formula | $C_{43}H_{54}F_6FeNO_8P$ | | | | | |
| molecular weight | 913.69 | | | | | |
| crystal system | Triclinic | | | | | |
| space group | P1bar (No.2) | | | | | |
| *a*/ Å | 10.3258(4) | 10.3393(4) | 10.3528(5) | 10.3641(5) | 10.3728(6) | 10.3843(6) |
| *b*/ Å | 11.1596(4) | 11.1764(4) | 11.195(5) | 11.2179(5) | 11.2401(6) | 11.2809(6) |
| *c*/ Å | 19.3998(8) | 19.3966(8) | 19.3916(8) | 19.3874(8) | 19.3827(10) | 19.3678(11) |
| $\alpha$/deg | 87.280(10) | 87.304(10) | 87.309(10) | 87.325(10) | 87.334(13) | 87.320(13) |
| $\beta$/deg | 79.573(9) | 79.839(9) | 80.133(9) | 80.422(9) | 80.672(12) | 81.11(12) |
| $\gamma$/deg | 88.854(10) | 88.826(9) | 88.751(10) | 88.710(10) | 88.679(12) | 88.583(13) |
| $V$/ Å$^3$ | 2195.93 | 2203.63 | 2211.58 | 2219.95 | 2227.28 | 2238.77 |
| $Z$ | 2 | 2 | 2 | 2 | 2 | 2 |
| $\mu$(MoKα)/ cm$^{-1}$ | 4.57 | 4.55 | 4.54 | 4.52 | 4.50 | 4.48 |
| $F$(000) | 956 | 956 | 956 | 956 | 956 | 956 |
| $D$/g cm$^{-3}$ | 1.382 | 1.377 | 1.372 | 1.367 | 1.362 | 1.355 |
| crystal size/mm | 0.30x0.22x 0.10 | 0.30x0.22x 0.10 | 0.30x0.22x 0.10 | 0.30x0.22x 0.10 | 0.30x0.22x 0.10 | 0.30x0.22x 0.10 |
| Unique reflections | 33063 | 33157 | 33225 | 33439 | 33557 | 33731 |
| Used reflections [$I$>2.0σ($I$)] | 9057 | 9091 | 9094 | 9145 | 9220 | 9234 |
| $R$ | 0.0517 | 0.0527 | 0.0540 | 0.0558 | 0.0565 | 0.0585 |
| $R$w | 0.1334 | 0.1381 | 0.1407 | 0.1447 | 0.1511 | 0.1536 |
| GOF | 1.040 | 1.042 | 1.041 | 1.045 | 1.047 | 1.025 |
